# 4-[3-(Benzyl­amino)-2-hy­droxy­prop­yl]-2,6-di-*tert*-butyl­phenol

**DOI:** 10.1107/S1600536811027115

**Published:** 2011-07-13

**Authors:** Ayten R. Asgarova, Mirze A. Allahverdiyev, Ali. N. Khalilov, Atash V. Gurbanov, Iván Brito

**Affiliations:** a Department of Organic Chemistry, Baku State University, Baku, Azerbaijan; b Departamento de Química, Facultad de Ciencias Básicas, Universidad de Antofagasta, Casilla 170, Antofagasta, Chile

## Abstract

In the title compound, C_24_H_35_NO_2_, the planes of the two aromatic rings form a dihedral angle of 72.76 (4)°. In the crystal, mol­ecules are linked by O—H⋯O and O—H⋯N hydrogen-bond inter­actions, forming an extended two-dimensional framework parallel to the *ab* plane.

## Related literature

For related compounds see: Asgarova *et al.* (2011 ▶); Krysin *et al.* (2010 ▶)
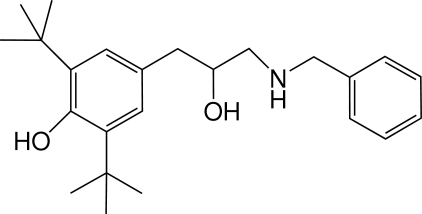


## Experimental

### 

#### Crystal data

C_24_H_35_NO_2_
*M* *_r_* = 369.53
Orthorhombic, 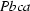

*a* = 9.8053 (10) Å
*b* = 18.870 (2) Å
*c* = 23.584 (3) Å
*V* = 4363.7 (8) Å^3^
*Z* = 8
Mo *K*α radiation
μ = 0.07 mm^−1^
*T* = 296 K
0.30 × 0.20 × 0.20 mm

#### Data collection

Bruker APEXII CCD diffractometer
Absorption correction: multi-scan (*SADABS*; Sheldrick, 2003 ▶) *T*_min_ = 0.979, *T*_max_ = 0.986
42619 measured reflections
4758 independent reflections
3478 reflections with *I* > 2σ(*I*)
*R*_int_ = 0.113

#### Refinement

*R*[*F* ^2^ > 2σ(*F* ^2^)] = 0.091
*wR*(*F* ^2^) = 0.243
*S* = 1.00
4758 reflections
250 parameters
H-atom parameters constrained
Δρ_max_ = 0.21 e Å^−3^
Δρ_min_ = −0.23 e Å^−3^

### 

Data collection: *APEX2* (Bruker, 2005 ▶); cell refinement: *SAINT-Plus* (Bruker, 2005 ▶); data reduction: *SAINT-Plus*; program(s) used to solve structure: *SHELXTL* (Sheldrick, 2008 ▶); program(s) used to refine structure: *SHELXTL*; molecular graphics: *SHELXTL*; software used to prepare material for publication: *WinGX* (Farrugia, 1997 ▶).

## Supplementary Material

Crystal structure: contains datablock(s) I, global. DOI: 10.1107/S1600536811027115/bt5572sup1.cif

Structure factors: contains datablock(s) I. DOI: 10.1107/S1600536811027115/bt5572Isup2.hkl

Supplementary material file. DOI: 10.1107/S1600536811027115/bt5572Isup3.cml

Additional supplementary materials:  crystallographic information; 3D view; checkCIF report

## Figures and Tables

**Table 1:** Hydrogen-bond geometry (Å, °)

*D*—H⋯*A*	*D*—H	H⋯*A*	*D*⋯*A*	*D*—H⋯*A*
O1—H1*O*⋯O2^i^	0.94	1.96	2.811 (3)	149
O2—H2*O*⋯N1^ii^	0.93	2.01	2.873 (4)	155

Symmetry codes: (i) 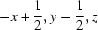; (ii) 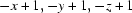.

**Fig. 1.**
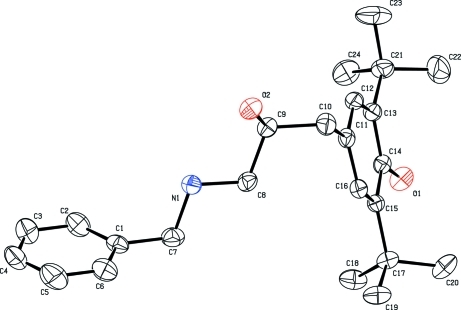
The structure of (I), showing the atom-numbering scheme. H atom were omitted for clarity. Displacement ellipsoids are drawn at the 30% probability level.

**Fig. 2.**
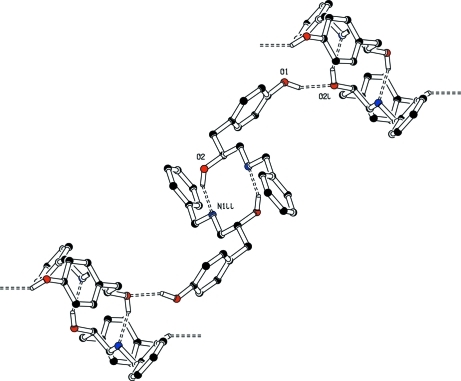
A view of (I) showing the two-dimensional framework constructed via O—H··· O and O—H··· N hydrogen bonds. Hydrogen bonds are depicted as dashed lines [symmetry code: (i) -x + 1/2, y - 1/2, z; (ii) -x + 1,-y + 1,-z + 1]. The tert-butyl groups and all H atoms not involved in hydrogen bonds were omitted for clarity

## supplementary crystallographic information

### Comment

Fig. 1 shows the molecular structure of title compound, (I). The benzene ring is coplanar with four and two of C and H atoms of the *tert*-butyl groups respectively, and with the O atom of the hydroxyl group on the aliphatic chain [r.m.s 0.0068 Å] and make a dihedral angle of 72.76 (4)° with the phenyl ring. In the crystal structure the molecules are linked by O—H···O and O—H···N hydrogen bond interactions forming an extended two-dimensional framework parallel to the *ab* plane, Fig. 2. In the literature, pharmaceutical activity of other similar compounds such as di-3-(3,5-di-*tert*-butyl-4-hydroxyphnyl)-2-hydroxy-1-(*N*-isopopylamino)propanesuccinate (Krysin *et al.*, 2010) has been reported.

### Experimental

A mixture of 2,6-di-*tert*-butyl-4-(3-chloro-2-hydroxypropyl)phenol (0.2 g, 0.00067 mol), with benzylamine (0.2 g, 0.0019 mol) and 0.1 g NaOH (0.1 g, 0.025 mol) was stirred in water (10 ml) for 8 h at 373K. After cooling down, the organic layer which was unsoluble in water easily separated. Then this solid crude product was crystallized in isopropanole. After recrystallizing in DMSO  single crystals were obtained. Yield 0.2g (80.97%). MP 135 °C. ^1^H NMR (300 MHz, DMSO-d_6_) δ 1.35 (s, 18H, 2(CH_3_)_3_), 1.98 (s, 1H, NH), 2.49 (d, 2H, CH_2_Ar),3.43 (s, 1H, OH), 3.67 (s, 4H, 2CH_2_N), 4.62 (s, 1H, OH), 6.91 (s, 2H, 2CH_arom_), 7.27(s, 5H, 5CH_arom_). ^13^CNMR (75 MHz, DMSO-d_6_) δ 30.90, 34.83,42.11, 53.63, 54.92, 71.36, 125.79, 126.92, 128.40, 128.52, 130.75, 139.21,141.34, 152.29

### Refinement

H atoms bonded to O and N were found in a difference map, but refined using a riding model starting from this position. All H-atoms bonded to C were placed in calculated positions [C—H = 0.99–0.93 Å, *U*_iso_(H) = 1.5 *U*_eq_(CH_3_) and *U*_iso_(H) = 1.2 *U*_eq_(CH and CH_2_] and were included in the refinement in the riding model approximation.

### Crystal data

**Table:**

C_24_H_35_NO_2_	*F*(000) = 1616
*M**_r_* = 369.53	*D*_x_ = 1.125 Mg m^−^^3^
Orthorhombic, *P**b**c**a*	Mo *K*α radiation, λ = 0.71073 Å
Hall symbol: -P 2ac 2ab	Cell parameters from 7811 reflections
*a* = 9.8053 (10) Å	θ = 2.3–26.2°
*b* = 18.870 (2) Å	µ = 0.07 mm^−^^1^
*c* = 23.584 (3) Å	*T* = 296 K
*V* = 4363.7 (8) Å^3^	Prism, colourless
*Z* = 8	0.30 × 0.20 × 0.20 mm

### Data collection

**Table:**

Bruker APEXII CCD diffractometer	4758 independent reflections
Radiation source: fine-focus sealed tube	3478 reflections with *I* > 2σ(*I*)
graphite	*R*_int_ = 0.113
φ and ω scans	θ_max_ = 27.0°, θ_min_ = 2.2°
Absorption correction: multi-scan (*SADABS*; Sheldrick, 2003)	*h* = −12→12
*T*_min_ = 0.979, *T*_max_ = 0.986	*k* = −24→24
42619 measured reflections	*l* = −30→30

### Refinement

**Table:**

Refinement on *F*^2^	Primary atom site location: structure-invariant direct methods
Least-squares matrix: full	Secondary atom site location: difference Fourier map
*R*[*F*^2^ > 2σ(*F*^2^)] = 0.091	Hydrogen site location: difference Fourier map
*wR*(*F*^2^) = 0.243	H-atom parameters constrained
*S* = 1.00	*w* = 1/[σ^2^(*F*_o_^2^) + (0.080*P*)^2^ + 7.4283*P*] where *P* = (*F*_o_^2^ + 2*F*_c_^2^)/3
4758 reflections	(Δ/σ)_max_ < 0.001
250 parameters	Δρ_max_ = 0.21 e Å^−^^3^
0 restraints	Δρ_min_ = −0.23 e Å^−^^3^

### Special details

**Table:**

Geometry. All e.s.d.'s (except the e.s.d. in the dihedral angle between two l.s. planes) are estimated using the full covariance matrix. The cell e.s.d.'s are taken into account individually in the estimation of e.s.d.'s in distances, angles and torsion angles; correlations between e.s.d.'s in cell parameters are only used when they are defined by crystal symmetry. An approximate (isotropic) treatment of cell e.s.d.'s is used for estimating e.s.d.'s involving l.s. planes.
Refinement. Refinement of *F*^2^ against ALL reflections. The weighted *R*-factor *wR* and goodness of fit *S* are based on *F*^2^, conventional *R*-factors *R* are based on *F*, with *F* set to zero for negative *F*^2^. The threshold expression of *F*^2^ > σ(*F*^2^) is used only for calculating *R*-factors(gt) *etc*. and is not relevant to the choice of reflections for refinement. *R*-factors based on *F*^2^ are statistically about twice as large as those based on *F*, and *R*- factors based on ALL data will be even larger.

### Fractional atomic coordinates and isotropic or equivalent isotropic displacement parameters (Å^2^)

**Table:**

	*x*	*y*	*z*	*U*_iso_*/*U*_eq_	
O1	0.0783 (2)	0.17031 (12)	0.38558 (11)	0.0539 (6)	
H1O	0.1199	0.1281	0.3981	0.081*	
O2	0.3869 (2)	0.54317 (11)	0.44598 (10)	0.0494 (6)	
H2O	0.4808	0.5481	0.4444	0.074*	
N1	0.3259 (3)	0.48697 (14)	0.55264 (11)	0.0423 (6)	
H1N	0.3293	0.5355	0.5496	0.051*	
C1	0.3085 (4)	0.50627 (18)	0.65547 (14)	0.0480 (8)	
C2	0.4181 (4)	0.4740 (2)	0.68157 (17)	0.0638 (10)	
H2A	0.4528	0.4319	0.6670	0.077*	
C3	0.4768 (5)	0.5039 (3)	0.72923 (18)	0.0777 (13)	
H3A	0.5519	0.4824	0.7461	0.093*	
C4	0.4248 (5)	0.5646 (3)	0.75143 (18)	0.0820 (14)	
H4A	0.4623	0.5837	0.7843	0.098*	
C5	0.3186 (6)	0.5974 (3)	0.7259 (2)	0.0935 (16)	
H5A	0.2850	0.6397	0.7406	0.112*	
C6	0.2600 (5)	0.5680 (3)	0.67820 (18)	0.0720 (12)	
H6A	0.1864	0.5906	0.6612	0.086*	
C7	0.2441 (4)	0.4746 (2)	0.60383 (15)	0.0557 (9)	
H7A	0.2332	0.4240	0.6094	0.067*	
H7B	0.1541	0.4949	0.5986	0.067*	
C8	0.2504 (3)	0.46587 (18)	0.50201 (13)	0.0447 (7)	
H8A	0.1696	0.4952	0.4985	0.054*	
H8B	0.2210	0.4170	0.5061	0.054*	
C9	0.3352 (3)	0.47289 (16)	0.44903 (13)	0.0407 (7)	
H9A	0.4125	0.4401	0.4518	0.049*	
C10	0.2549 (4)	0.45515 (19)	0.39529 (14)	0.0508 (8)	
H10A	0.1756	0.4857	0.3934	0.061*	
H10B	0.3115	0.4655	0.3626	0.061*	
C11	0.2085 (3)	0.37951 (17)	0.39179 (12)	0.0394 (7)	
C12	0.2873 (3)	0.32842 (18)	0.36563 (13)	0.0438 (7)	
H12A	0.3699	0.3420	0.3496	0.053*	
C13	0.2495 (3)	0.25828 (18)	0.36215 (13)	0.0424 (7)	
C14	0.1253 (3)	0.23858 (16)	0.38807 (12)	0.0373 (6)	
C15	0.0408 (3)	0.28881 (15)	0.41439 (12)	0.0346 (6)	
C16	0.0847 (3)	0.35827 (16)	0.41475 (12)	0.0380 (7)	
H16A	0.0289	0.3924	0.4311	0.046*	
C17	−0.0959 (3)	0.26783 (17)	0.44140 (14)	0.0447 (7)	
C18	−0.0733 (4)	0.2144 (2)	0.48945 (18)	0.0654 (11)	
H18A	−0.1587	0.2044	0.5077	0.098*	
H18B	−0.0106	0.2338	0.5166	0.098*	
H18C	−0.0363	0.1713	0.4741	0.098*	
C19	−0.1680 (4)	0.3316 (2)	0.46782 (19)	0.0654 (11)	
H19A	−0.2506	0.3162	0.4859	0.098*	
H19B	−0.1893	0.3654	0.4388	0.098*	
H19C	−0.1093	0.3531	0.4955	0.098*	
C20	−0.1917 (4)	0.2370 (2)	0.3965 (2)	0.0698 (12)	
H20A	−0.2779	0.2257	0.4137	0.105*	
H20B	−0.1521	0.1947	0.3809	0.105*	
H20C	−0.2052	0.2711	0.3669	0.105*	
C21	0.3381 (4)	0.2040 (2)	0.33008 (16)	0.0582 (10)	
C22	0.2571 (5)	0.1687 (3)	0.2829 (2)	0.105 (2)	
H22A	0.3158	0.1376	0.2618	0.157*	
H22B	0.2208	0.2043	0.2581	0.157*	
H22C	0.1836	0.1418	0.2991	0.157*	
C23	0.4598 (5)	0.2401 (3)	0.3017 (3)	0.106 (2)	
H23A	0.5140	0.2052	0.2825	0.158*	
H23B	0.5141	0.2634	0.3300	0.158*	
H23C	0.4276	0.2744	0.2748	0.158*	
C24	0.3973 (5)	0.1493 (3)	0.3702 (2)	0.0795 (13)	
H24A	0.4552	0.1175	0.3494	0.119*	
H24B	0.3245	0.1229	0.3875	0.119*	
H24C	0.4496	0.1726	0.3990	0.119*	

### Atomic displacement parameters (Å^2^)

**Table:**

	*U*^11^	*U*^22^	*U*^33^	*U*^12^	*U*^13^	*U*^23^
O1	0.0534 (13)	0.0337 (12)	0.0747 (16)	0.0011 (10)	−0.0039 (12)	0.0016 (11)
O2	0.0490 (12)	0.0341 (12)	0.0651 (15)	−0.0086 (10)	0.0005 (11)	0.0037 (10)
N1	0.0461 (14)	0.0367 (14)	0.0442 (14)	−0.0042 (11)	0.0033 (11)	−0.0043 (11)
C1	0.0559 (19)	0.0449 (19)	0.0433 (17)	−0.0066 (15)	0.0131 (15)	0.0010 (15)
C2	0.082 (3)	0.054 (2)	0.056 (2)	0.004 (2)	0.005 (2)	−0.0031 (18)
C3	0.081 (3)	0.097 (4)	0.055 (2)	−0.005 (3)	−0.004 (2)	0.002 (2)
C4	0.089 (3)	0.114 (4)	0.044 (2)	−0.020 (3)	0.008 (2)	−0.017 (3)
C5	0.106 (4)	0.101 (4)	0.074 (3)	0.008 (3)	0.017 (3)	−0.035 (3)
C6	0.075 (3)	0.079 (3)	0.063 (2)	0.009 (2)	0.011 (2)	−0.016 (2)
C7	0.0503 (18)	0.060 (2)	0.057 (2)	−0.0098 (17)	0.0099 (17)	0.0030 (18)
C8	0.0410 (15)	0.0418 (17)	0.0514 (18)	−0.0083 (14)	−0.0009 (14)	−0.0021 (15)
C9	0.0427 (16)	0.0295 (15)	0.0500 (17)	−0.0063 (12)	−0.0011 (13)	0.0042 (13)
C10	0.0574 (19)	0.049 (2)	0.0456 (18)	−0.0123 (16)	−0.0049 (16)	0.0118 (15)
C11	0.0428 (16)	0.0410 (17)	0.0344 (14)	−0.0074 (13)	−0.0057 (12)	0.0083 (13)
C12	0.0372 (15)	0.057 (2)	0.0375 (15)	−0.0035 (14)	0.0003 (12)	0.0092 (15)
C13	0.0374 (14)	0.0522 (19)	0.0377 (15)	0.0077 (14)	0.0001 (13)	0.0034 (14)
C14	0.0381 (14)	0.0344 (16)	0.0395 (15)	0.0004 (12)	−0.0042 (12)	0.0053 (12)
C15	0.0354 (14)	0.0348 (15)	0.0335 (14)	0.0006 (12)	−0.0003 (11)	0.0029 (12)
C16	0.0418 (15)	0.0363 (16)	0.0359 (14)	0.0020 (13)	−0.0020 (12)	−0.0006 (12)
C17	0.0361 (15)	0.0415 (18)	0.0563 (19)	−0.0004 (13)	0.0069 (14)	0.0008 (15)
C18	0.064 (2)	0.063 (3)	0.069 (2)	0.0023 (19)	0.021 (2)	0.012 (2)
C19	0.051 (2)	0.067 (3)	0.078 (3)	0.0075 (19)	0.0229 (19)	0.002 (2)
C20	0.0408 (18)	0.068 (3)	0.101 (3)	−0.0070 (18)	−0.009 (2)	−0.005 (2)
C21	0.0445 (18)	0.073 (3)	0.057 (2)	0.0152 (18)	0.0074 (16)	−0.0003 (19)
C22	0.083 (3)	0.157 (6)	0.075 (3)	0.041 (4)	−0.010 (3)	−0.052 (3)
C23	0.074 (3)	0.128 (5)	0.115 (4)	0.025 (3)	0.051 (3)	0.007 (4)
C24	0.062 (2)	0.084 (3)	0.093 (3)	0.031 (2)	−0.002 (2)	−0.001 (3)

### Geometric parameters (Å, °)

**Table:**

O1—C14	1.370 (4)	C12—H12A	0.9300
O1—H1O	0.9421	C13—C14	1.412 (4)
O2—C9	1.422 (4)	C13—C21	1.541 (5)
O2—H2O	0.9261	C14—C15	1.404 (4)
N1—C8	1.460 (4)	C15—C16	1.380 (4)
N1—C7	1.468 (4)	C15—C17	1.536 (4)
N1—H1N	0.9181	C16—H16A	0.9300
C1—C6	1.368 (5)	C17—C19	1.528 (5)
C1—C2	1.381 (5)	C17—C20	1.530 (5)
C1—C7	1.496 (5)	C17—C18	1.533 (5)
C2—C3	1.383 (6)	C18—H18A	0.9600
C2—H2A	0.9300	C18—H18B	0.9600
C3—C4	1.359 (7)	C18—H18C	0.9600
C3—H3A	0.9300	C19—H19A	0.9600
C4—C5	1.352 (7)	C19—H19B	0.9600
C4—H4A	0.9300	C19—H19C	0.9600
C5—C6	1.380 (6)	C20—H20A	0.9600
C5—H5A	0.9300	C20—H20B	0.9600
C6—H6A	0.9300	C20—H20C	0.9600
C7—H7A	0.9700	C21—C24	1.516 (6)
C7—H7B	0.9700	C21—C22	1.521 (6)
C8—C9	1.507 (4)	C21—C23	1.528 (6)
C8—H8A	0.9700	C22—H22A	0.9600
C8—H8B	0.9700	C22—H22B	0.9600
C9—C10	1.529 (4)	C22—H22C	0.9600
C9—H9A	0.9800	C23—H23A	0.9600
C10—C11	1.500 (5)	C23—H23B	0.9600
C10—H10A	0.9700	C23—H23C	0.9600
C10—H10B	0.9700	C24—H24A	0.9600
C11—C12	1.381 (5)	C24—H24B	0.9600
C11—C16	1.388 (4)	C24—H24C	0.9600
C12—C13	1.377 (5)		
			
C14—O1—H1O	129.5	O1—C14—C13	121.3 (3)
C9—O2—H2O	116.8	C15—C14—C13	121.5 (3)
C8—N1—C7	110.6 (2)	C16—C15—C14	117.4 (3)
C8—N1—H1N	103.2	C16—C15—C17	121.0 (3)
C7—N1—H1N	104.0	C14—C15—C17	121.7 (3)
C6—C1—C2	118.1 (4)	C15—C16—C11	123.0 (3)
C6—C1—C7	120.9 (4)	C15—C16—H16A	118.5
C2—C1—C7	121.0 (3)	C11—C16—H16A	118.5
C1—C2—C3	120.4 (4)	C19—C17—C20	107.3 (3)
C1—C2—H2A	119.8	C19—C17—C18	106.5 (3)
C3—C2—H2A	119.8	C20—C17—C18	110.4 (3)
C4—C3—C2	120.1 (5)	C19—C17—C15	111.7 (3)
C4—C3—H3A	120.0	C20—C17—C15	110.3 (3)
C2—C3—H3A	120.0	C18—C17—C15	110.5 (3)
C5—C4—C3	120.2 (4)	C17—C18—H18A	109.5
C5—C4—H4A	119.9	C17—C18—H18B	109.5
C3—C4—H4A	119.9	H18A—C18—H18B	109.5
C4—C5—C6	119.9 (5)	C17—C18—H18C	109.5
C4—C5—H5A	120.0	H18A—C18—H18C	109.5
C6—C5—H5A	120.0	H18B—C18—H18C	109.5
C1—C6—C5	121.2 (5)	C17—C19—H19A	109.5
C1—C6—H6A	119.4	C17—C19—H19B	109.5
C5—C6—H6A	119.4	H19A—C19—H19B	109.5
N1—C7—C1	112.0 (3)	C17—C19—H19C	109.5
N1—C7—H7A	109.2	H19A—C19—H19C	109.5
C1—C7—H7A	109.2	H19B—C19—H19C	109.5
N1—C7—H7B	109.2	C17—C20—H20A	109.5
C1—C7—H7B	109.2	C17—C20—H20B	109.5
H7A—C7—H7B	107.9	H20A—C20—H20B	109.5
N1—C8—C9	112.0 (2)	C17—C20—H20C	109.5
N1—C8—H8A	109.2	H20A—C20—H20C	109.5
C9—C8—H8A	109.2	H20B—C20—H20C	109.5
N1—C8—H8B	109.2	C24—C21—C22	110.9 (4)
C9—C8—H8B	109.2	C24—C21—C23	106.1 (4)
H8A—C8—H8B	107.9	C22—C21—C23	106.4 (4)
O2—C9—C8	108.7 (3)	C24—C21—C13	111.3 (3)
O2—C9—C10	110.2 (3)	C22—C21—C13	110.8 (3)
C8—C9—C10	112.6 (3)	C23—C21—C13	111.1 (4)
O2—C9—H9A	108.4	C21—C22—H22A	109.5
C8—C9—H9A	108.4	C21—C22—H22B	109.5
C10—C9—H9A	108.4	H22A—C22—H22B	109.5
C11—C10—C9	114.2 (3)	C21—C22—H22C	109.5
C11—C10—H10A	108.7	H22A—C22—H22C	109.5
C9—C10—H10A	108.7	H22B—C22—H22C	109.5
C11—C10—H10B	108.7	C21—C23—H23A	109.5
C9—C10—H10B	108.7	C21—C23—H23B	109.5
H10A—C10—H10B	107.6	H23A—C23—H23B	109.5
C12—C11—C16	117.5 (3)	C21—C23—H23C	109.5
C12—C11—C10	121.3 (3)	H23A—C23—H23C	109.5
C16—C11—C10	121.2 (3)	H23B—C23—H23C	109.5
C13—C12—C11	123.2 (3)	C21—C24—H24A	109.5
C13—C12—H12A	118.4	C21—C24—H24B	109.5
C11—C12—H12A	118.4	H24A—C24—H24B	109.5
C12—C13—C14	117.4 (3)	C21—C24—H24C	109.5
C12—C13—C21	121.1 (3)	H24A—C24—H24C	109.5
C14—C13—C21	121.6 (3)	H24B—C24—H24C	109.5
O1—C14—C15	117.1 (3)		
			
C6—C1—C2—C3	−0.2 (6)	C21—C13—C14—O1	−0.2 (4)
C7—C1—C2—C3	179.9 (4)	C12—C13—C14—C15	2.5 (4)
C1—C2—C3—C4	1.4 (7)	C21—C13—C14—C15	−176.2 (3)
C2—C3—C4—C5	−2.3 (7)	O1—C14—C15—C16	−177.1 (3)
C3—C4—C5—C6	2.0 (8)	C13—C14—C15—C16	−1.0 (4)
C2—C1—C6—C5	−0.2 (6)	O1—C14—C15—C17	2.4 (4)
C7—C1—C6—C5	179.8 (4)	C13—C14—C15—C17	178.5 (3)
C4—C5—C6—C1	−0.7 (8)	C14—C15—C16—C11	−1.7 (4)
C8—N1—C7—C1	−170.7 (3)	C17—C15—C16—C11	178.8 (3)
C6—C1—C7—N1	103.3 (4)	C12—C11—C16—C15	2.6 (4)
C2—C1—C7—N1	−76.8 (4)	C10—C11—C16—C15	−177.4 (3)
C7—N1—C8—C9	−175.8 (3)	C16—C15—C17—C19	−1.2 (4)
N1—C8—C9—O2	−54.2 (3)	C14—C15—C17—C19	179.3 (3)
N1—C8—C9—C10	−176.6 (3)	C16—C15—C17—C20	118.1 (3)
O2—C9—C10—C11	173.9 (3)	C14—C15—C17—C20	−61.4 (4)
C8—C9—C10—C11	−64.5 (4)	C16—C15—C17—C18	−119.5 (3)
C9—C10—C11—C12	−91.6 (4)	C14—C15—C17—C18	61.0 (4)
C9—C10—C11—C16	88.4 (4)	C12—C13—C21—C24	114.3 (4)
C16—C11—C12—C13	−0.9 (4)	C14—C13—C21—C24	−67.0 (4)
C10—C11—C12—C13	179.1 (3)	C12—C13—C21—C22	−121.8 (4)
C11—C12—C13—C14	−1.6 (5)	C14—C13—C21—C22	56.9 (5)
C11—C12—C13—C21	177.2 (3)	C12—C13—C21—C23	−3.8 (5)
C12—C13—C14—O1	178.5 (3)	C14—C13—C21—C23	174.9 (4)

### Hydrogen-bond geometry (Å, °)

**Table:**

*D*—H···*A*	*D*—H	H···*A*	*D*···*A*	*D*—H···*A*
O1—H1O···O2^i^	0.94	1.96	2.811 (3)	149
O2—H2O···N1^ii^	0.93	2.01	2.873 (4)	155

Symmetry codes: (i) −*x*+1/2, *y*−1/2, *z*; (ii) −*x*+1, −*y*+1, −*z*+1.
